# Chloroform Extract of* Artemisia annua *L. Relaxes Mouse Airway Smooth Muscle

**DOI:** 10.1155/2017/9870414

**Published:** 2017-11-12

**Authors:** Jun Huang, Li-Qun Ma, Yongle Yang, Nana Wen, Wan Zhou, Congli Cai, Qing-hua Liu, Jinhua Shen

**Affiliations:** ^1^Institute for Medical Biology and Hubei Provincial Key Laboratory for Protection and Application of Special Plants in Wuling Area of China, College of Life Sciences, South-Central University for Nationalities, Wuhan 430074, China; ^2^Wuhan Youzhiyou Biopharmaceutical Co., Ltd., 666 Gaoxin Rd., Biolake, Wuhan 430075, China

## Abstract

*Artemisia annua *L. belongs to the Asteraceae family, which is indigenous to China. It has valuable pharmacological properties, such as antimalarial, anti-inflammatory, and anticancer properties. However, whether it possesses antiasthma properties is unknown. In the current study, chloroform extract of* Artemisia annua *L. (CEAA) was prepared, and we found that CEAA completely eliminated acetylcholine (ACh) or high K^+^-elicited (80 mM) contractions of mouse tracheal rings (TRs). Patch-clamp technique and ion channel blockers were employed to explore the underlying mechanisms of the relaxant effect of CEAA. In whole-cell current recording, CEAA almost fully abolished voltage-dependent Ca^2+^ channel (VDCC) currents and markedly enhanced large conductance Ca^2+^-activated K^+^ (BK) channel currents on airway smooth muscle cells (ASMCs). In single channel current recording, CEAA increased the opening probability but had no effect on the single channel conductance of BK channels. However, under paxilline-preincubated (a selective BK channel blocker) conditions, CEAA only slightly increased BK channel currents. These results indicate that CEAA may contain active components with potent antiasthma activity. The abolished VDCCs by CEAA may mainly contribute to the underlying mechanism through which it acts as an effective antiasthmatic compound, but the enhanced BK currents might play a less important role in the antiasthmatic effects.

## 1. Introduction

Millions of people suffer from asthma worldwide; the prevalence of this disease is increasing along with the increase in air pollution [1]. According to the World Health Organization, asthma is becoming a major economic, social, and healthcare burden throughout the world and has been identified as a public health priority [2]. One of the characteristic features of asthma is hyperresponsiveness of airway smooth muscle (ASM) [3]. At present, the preferred first-line treatment for asthma is still *β*_2_ adrenergic receptor (*β*_2_ AR) agonists combined with glucocorticoids, but this therapeutic strategy causes multiple severe side-effects, including palpitations, tremors, headache, cardiovascular death, and cardiac failure [4–7]. Moreover, many patients who receive this treatment suffer from repeated asthmatic attacks [8]. Thus, it is necessary to develop novel drugs with increased efficacy but fewer side-effects for antiasthma treatments.

China has a long history of therapeutic application of botanical drugs in traditional medicine [9].* Artemisia annua *L. belongs to the Asteraceae family, and it has been extensively used for thousands years to treat multiple diseases, including malaria, fever, acanthamoebiasis, schistosomiasis, eczema, arthritis, and rheumatism [10, 11]. Artemisinin, a well-known antimalaria compound, was originally isolated from the plant* Artemisia annua *L. by Chinese scientists, and it is currently an established standard treatment for malaria worldwide. In addition to its well-known antimalaria effects, accumulating evidence confirmed that* Artemisia annua *L. also possesses a wide spectrum of other pharmacological activities, such as antibacterial, antiviral, anticancer, anti-inflammatory, and immunosuppressive effects [12–15]. More recently, several studies have shown that artemisinin has strong antioxidant activity [16]. However, the action of* Artemisia annua *L. on airway hyperreactivity is unknown. In the present study, tracheal rings (TRs) and acute isolated airway smooth muscle cells (ASMCs) of mice were used; high K^+^ (80 mM K^+^) and acetylcholine (ACh, 100 *μ*M) were employed to mimic airway hyperreactiveness to evaluate the potent antiasthma capability of* Artemisia annua *L. and explore the underlying mechanism. The results clearly indicated that CEAA effectively relaxed ACh-induced TRs contractions by inhibiting VDCC-mediated Ca^2+^ influx, thus exhibiting potent antiasthma activity.

## 2. Materials and Methods

### 2.1. Guideline Statement

All methods used in this study are in accordance with protocols approved by the South-Central University for Nationalities. All animal studies and experiments were conducted under guidelines and protocols approved by the Institutional Animal Care and Use Committee of the South-Central University for Nationalities. The experiments were performed under guidelines and protocols approved by the Ethics Committee of the South-Central University for Nationalities.

### 2.2. Reagents

Nifedipine, ACh, paxilline, bovine serum albumin (BSA), papain, collagenase H, dithiothreitol (DTT), CsOH, cesium chloride (CsCl), Mg-ATP, and TEA-Cl were purchased from Sigma (St. Louis, MO, USA). Nifedipine was dissolved in dimethyl sulfoxide (DMSO) and others in corresponding solutions used in the experiments.

### 2.3. Plant Material and Extraction

*Artemisia annua *L. was purchased from Beijing Tongrentang (Wuhan, China) and was authenticated by Professor Ding-Rong Wan (College of Pharmacy, South-Central University for Nationalities). A voucher specimen was deposited at the Herbarium of College of Pharmacy, South-Central University for Nationalities, China.

Briefly,* Artemisia annua *L. (500 g) was dried, powdered, and then soaked with 1 L ddH_2_O at 55°C for 4 hours. The extracts were filtered, and the supernatants were collected and evaporated under reduced pressure. The dried products were dissolved in 200 mL water, and the lipids were removed using petroleum ether. The remaining components were then extracted with chloroform and dried under reduced pressure. The final product, CEAA, was dissolved in 3% DMSO for all experiments.

### 2.4. Animals

Six-week-old male BALB/c mice were purchased from the Hubei Provincial Center for Disease Control and Prevention (Wuhan, China) and were housed in specific pathogen-free (SPF) grade laboratory rooms with 12 h light-12 h dark cycles.

### 2.5. Measurement of Tension

Mouse ASM contraction was measured in TRs as previously described [17]. Briefly, tracheas were isolated from cervical dislocated mice. TRs (~5 mm) from the bottom of the tracheae were cut and mounted in 10 mL organ baths containing PSS bubbled with 95% O_2_ and 5% CO_2_ at 37°C. The composition of PSS (mM) was as follows: NaCl 135, KCl 5, MgCl_2_ 1, CaCl_2_ 2, HEPES 10, and glucose 10 (pH 7.4, adjusted with NaOH). The resting tension was set to 0.3 g. The experiment was initiated after the TRs were equilibrated for 60 min. Then, the TRs were stimulated with either 80 mM K^+^ or 100 *μ*M ACh 3 times. Following an additional 30 min rest, the isometric tension was measured under high K^+^, ACh, or CEAA. The high K^+^ solutions contained (mM) NaCl 60, KCl 80, MgCl_2_ 1, CaCl_2_ 2, HEPES 10, and glucose 10 (pH 7.4 with NaOH).

### 2.6. Recording of VDCC Currents

Mouse ASM cells were isolated as previously described [17]. Whole-cell Ca^2+^ currents through VDCC were recorded using an EPC-10 patch-clamp amplifier (HEKA, Lambrecht, Germany). The pipette solution containing 130 mM CsCl, 10 mM EGTA, 10 mM HEPES, 4 mM Mg-ATP, and 4 mM MgCl_2_ was adjusted to pH 7.2 with CsOH. The composition of the external solution was 27.5 mM BaCl_2_, 11 mM glucose, 10 mM HEPES, 1 mM MgCl_2_, 107 mM NaCl, and 10 mM TEA-Cl, and it was adjusted to pH 7.4 with NaOH. The membrane potential of ASMCs was held at −70 mV. The currents were elicited following step depolarization for 1000 msec from −70 to +40 mV with 10 mV increments with a 30 msec interval.

### 2.7. Recording of BK Currents

In whole-cell recording, depolarizing voltage steps with 10 mV increments were applied from a holding potential of −80 mV to +80 mV to elicit BK currents. The pipette solution containing 10 mM NaCl, 125 mM KCl, 10 mM EGTA, 10 mM HEPES, and 6.2 mM MgCl_2_ was adjusted to pH 7.2 with KOH. The bath solution containing 5.4 mM KCl, 150 mM NaCl, 5.4 mM CaCl_2_, 0.8 mM MgCl_2_, and 10 mM HEPES was adjusted to pH 7.2 with KOH.

Single BK currents were recorded at 0, 20, 40, and 60 mV using outside-out techniques under symmetrical K^+^ ion concentrations in the pipette and bath solutions. The pipette solution containing 4.37 mM CaCl_2_, 140 mM KCl, 5 mM EGTA, 10 mM HEPES, and 1 mM MgCl_2_ was adjusted to pH 7.2 with KOH. The bath solution containing 4.9 mM CaCl_2_, 140 mM KCl, 1 mM MgCl_2_, 10 mM HEPES, and 1 mM EGTA was adjusted to pH 7.2 with KOH. Single channel currents were acquired at a digitization rate of 4 kHz and filtered at 1 kHz. Events were detected and all-point amplitude histogram and single channel open probability (Po) obtained using Clampfit 9 software (Axon Instruments; Foster City, CA, USA).

### 2.8. Statistical Analysis

Statistical analysis and significance were measured with Student's *t*-test using Origin 9.0 software (OriginLab, Northampton, USA). The results are expressed as the mean ± SEM. In all comparisons, *p* < 0.05 was considered statistically significant.

## 3. Results

### 3.1. CEAA Dose-Dependently Inhibited High K^+^-Induced TRs Contraction

**Figure 1:**
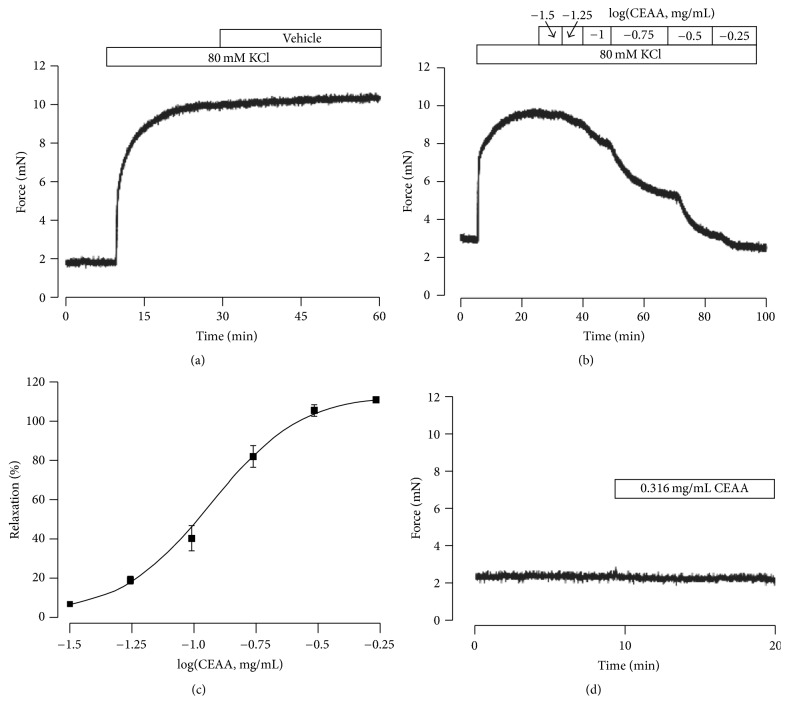
CEAA inhibits high K^+^-induced contraction of ASM in mice. (a) High K^+^ induced a sustainable contraction in a tracheal ring. (b) High K^+^-induced contraction was gradually relaxed by cumulative administration of CEAA. (c) The experiment was repeated in 9 TRs/9 mice, and a dose-relaxation curve was plotted. The IC_50_ of CEAA was 0.316 mg/ml. (d) CEAA had no effects on the basic tone of ASM.

Extracellular high K^+^- (80 mM [K^+^]_o_-) induced contraction of smooth muscles was extensively verified [18]. As shown in Figure 1(a), we successfully recorded the high K^+^-induced sustainable contractions. We then observed that CEAA inhibited the high K^+^-induced contraction in a dose-dependent manner on mouse TRs. As shown in Figure 1(b), the maximal relaxation produced by CEAA reached 100% (*n* = 8). The half-maximal inhibition (IC_50_) of CEAA was 0.316 mg/ml (Figure 1(c)). After washing out of CEAA, the high K^+^-induced contraction recover by 35.9%  ±  3.5% (see Figure S1A in Supplementary Material available online at https://doi.org/10.1155/2017/9870414). This result indicated that CEAA exerted a long-term effect on high K^+^-induced contractions of ASM. However, CEAA had no effect on the resting tone of TRs (Figure 1(d)).

### 3.2. CEAA Decreased High K^+^-Evoked Ca^2+^ Influx by Blocking VDCC Currents

**Figure 2:**
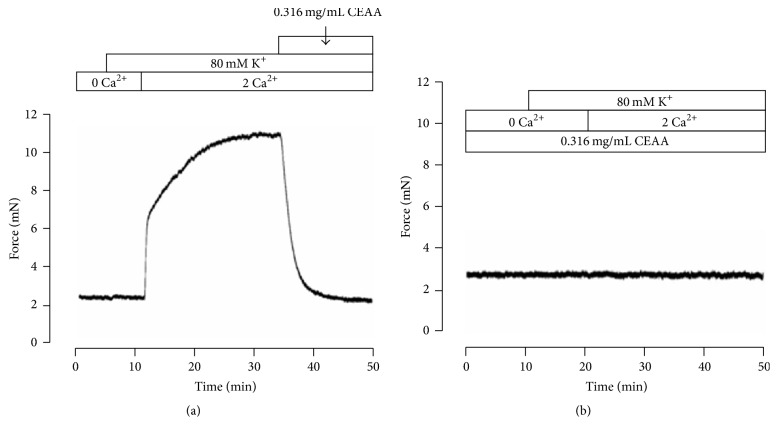
CEAA blocks high K^+^-evoked Ca^2+^ influx. (a) A representative force tracing of 4 TRs/4 mice. Under Ca^2+^-free conditions (0 Ca^2+^ and 0.5 mM EGTA), high K^+^ failed to elicit contraction in a tracheal ring. Following restoration of 2 mM Ca^2+^, a sustained contraction occurred, and it was completely inhibited by 0.316 mg/ml CEAA. (b) Identical experiments were performed as described in the presence of 0.316 mg/ml CEAA, and high K^+^-induced contraction did not appear after the restoration of 2 mM Ca^2+^.

When elevating extracellular K^+^ concentration, the membrane potential of cells depolarizes, which opens VDCCs, resulting in Ca^2+^ influx and triggering ASM contraction [19]. Whether CEAA inhibited the high K^+^-induced contraction by blocking this pathway is unknown. We first tested this hypothesis by depriving cells of and subsequently restoring extracellular Ca^2+^. As shown in Figure 2(a), under Ca^2+^-free conditions (0 mM Ca^2+^ and 0.5 mM EGTA), 80 mM K^+^ did not induce contractions in TRs, but the contractions appeared after restoration of the extracellular Ca^2+^ concentration to 2 mM. CEAA abolished the restored contractions almost completely. Furthermore, preincubation of TRs with CEAA also completely eliminated the high K^+^-induced contractions with replenished extracellular Ca^2+^ from 0 to 2 mM (Figure 2(b)).

**Figure 3:**
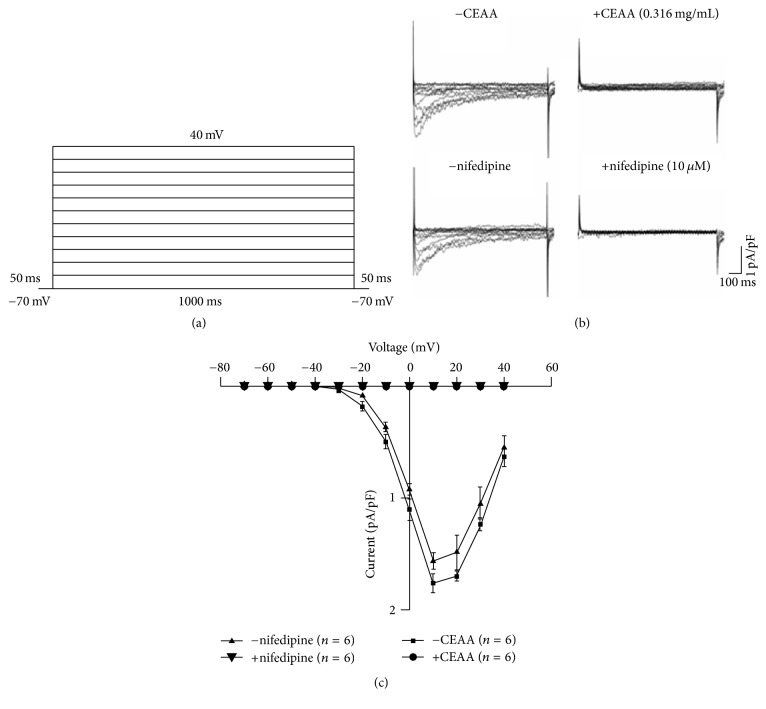
CEAA blocks VDCC currents on ASMCs. (a) The ASMC membrane under voltage clamp was stepped up from a holding potential of −70 mV to test-pulse potentials ranging in 10 mV steps from −60 mV to +40 mV. (b) VDCC currents were recorded following the depolarization steps as described in (a), and they were blocked by CEAA or nifedipine. (c)* I-V* relations were plotted based on the results of 7 experiments.

To further clarify the contribution of VDCCs on the relaxant effects of CEAA, we employed whole-cell patch clamp to record VDCC currents. As shown in Figure 3, the VDCC currents were elicited by step voltage depolarization from −70 mV to +40 mV with 10 mV increments (Figure 3(a)) and blocked by nifedipine (10 *μ*M), indicating that these are VDCC currents (Figure 3(b), bottom). We then observed the effects of CEAA on VDCC currents and found that the currents were abolished by CEAA (0.316 mg/ml) (Figure 3(b), top). Current-voltage (*I-V*) curves were plotted, and CEAA decreased the maximum amplitude of VDCC (*n* = 6, Figure 3(c)). These results suggested that CEAA may block Ca^2+^ influx by inhibiting VDCC currents, leading to relaxation.

### 3.3. CEAA Inhibited ACh-Induced Contraction in TRs

**Figure 4:**
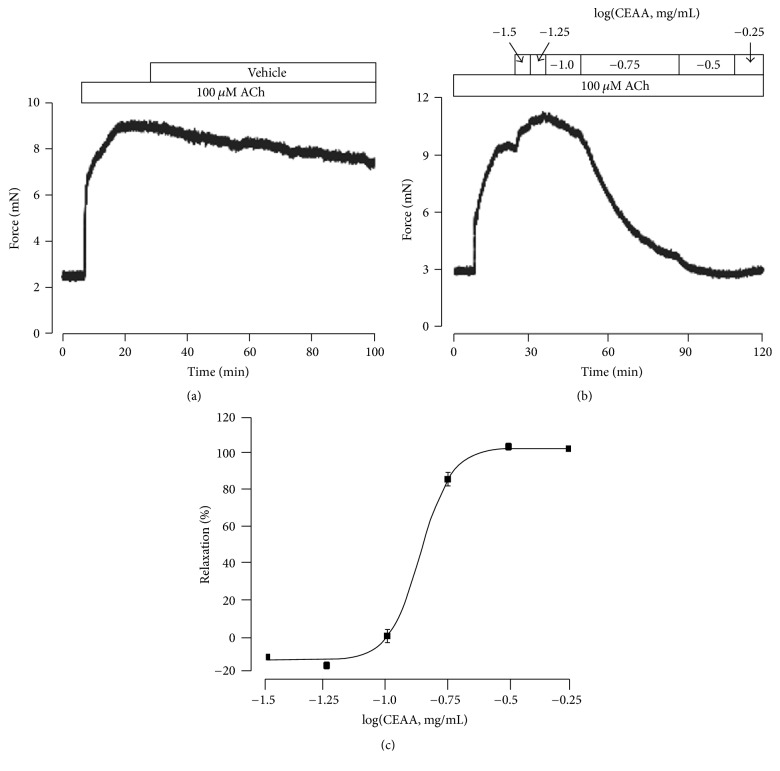
CEAA suppresses ACh-induced contraction of mouse TRs. (a) ACh induced a steady-state contraction in a mouse tracheal ring. (b) ACh-induced contraction was inhibited by CEAA in a concentration-dependent manner. (c) Dose-relaxation curve of CEAA based on the results of 7 different experiments was plotted.

ACh is one of the major contractile stimuli, and it determines the contractile state of airway smooth muscle [20]. We first recorded the stable and sustainable ACh-induced contractions on mouse TRs (Figure 4(a)). We then observed the effect of CEAA on ACh-induced contractions. As shown in Figure 4(b), CEAA inhibited ACh-induced contractions dose-dependently, and the dose-effect curve was plotted (Figure 4(c)). A brief application of 0.316 mg/ml CEAA fully abolished ACh-induced contractions with 45.8%  ±  1.2% recovery after washout (Figure S1 B).

**Figure 5:**
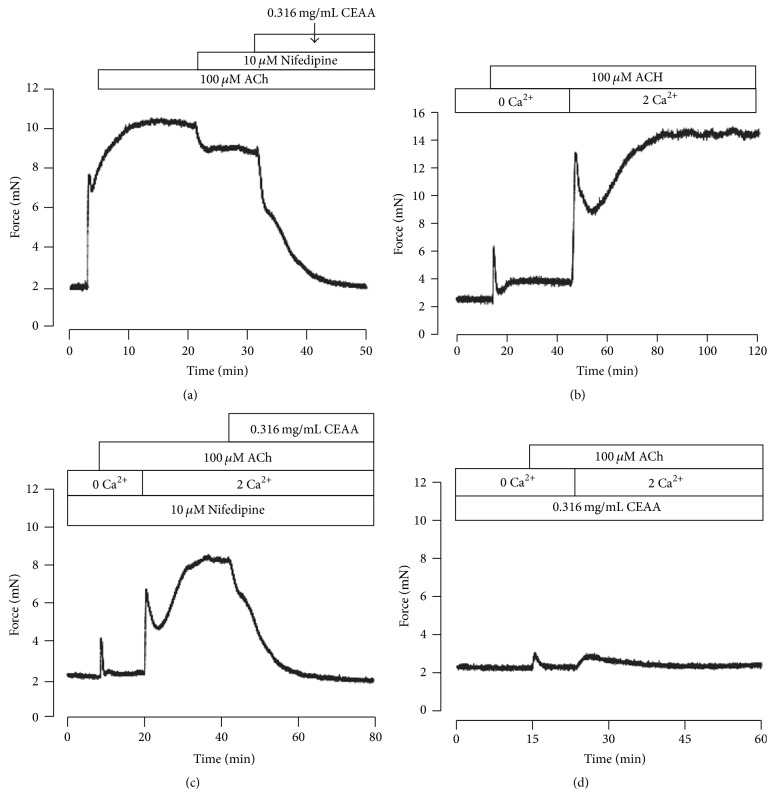
CEAA blocks ACh-elicited Ca^2+^ influx. (a) ACh induced contractions in TRs, which were inhibited in part by nifedipine, and the remaining contraction was inhibited by CEAA. (b) ACh induced a rapid and transient contraction under Ca^2+^-free conditions (0 Ca^2+^ and 0.5 mM EGTA); following the addition of 2 mM Ca^2+^, a strong sustained contraction occurred. (c) A set of experiments was performed in the presence of nifedipine (*n* = 4). Compared with (b), nifedipine had no effect on the transient contraction under 0 Ca^2+^ conditions but significantly decreased the sustained contraction under 2 mM Ca^2+^ conditions. (d) A set of experiments was conducted in the presence of CEAA (*n* = 4). Under Ca^2+^-free conditions (0 Ca^2+^ and 0.5 mM EGTA); ACh induced a smaller transient contraction than that with no CEAA. Following the addition of 2 mM Ca^2+^, only a very weak contraction occurred.

ACh induces the contraction of ASM through multiple pathways, including opening VDCCs and inhibiting the activity of BK channels [21]. As illustrated in Figure 5(a), ACh-induced contractions were inhibited in part by administration of nifedipine (10 *μ*M) and then eliminated completely by CEAA. To further confirm the contribution of VDCCs on CEAA-induced relaxation, a set of identical experiments were performed in the presence of nifedipine (10 *μ*M) or CEAA. As shown in Figure 5(b), under Ca^2+^-free conditions (0 Ca^2+^ and 0.5 mM EGTA), ACh induced a transient contraction; then, a sustained contraction occurred when recovering 2 mM Ca^2+^. When preincubation with 10 *μ*M nifedipine (Figure 5(c)), the amplitude of ACh-induced transient contractions under Ca^2+^-free conditions had no significant changes; but the amplitude of the sustained contractions under 2 mM Ca^2+^ conditions decreased markedly; subsequently, the contractions were blocked fully by CEAA. However, both the transient contractions under Ca^2+^-free conditions and the sustained contractions under 2 mM Ca^2+^ conditions were drastically decreased by preincubation of CEAA (Figure 5(d)). These data together suggested that VDCCs and the other pathways were involved in the relaxant effects of CEAA on ACh-induced contractions. Moreover, the transient contraction was blocked by CEAA, indicating that CEAA also attenuated the intracellular Ca^2+^ release.

### 3.4. K^+^ Channels Were Involved in the Relaxant Effects of CEAA

**Figure 6:**
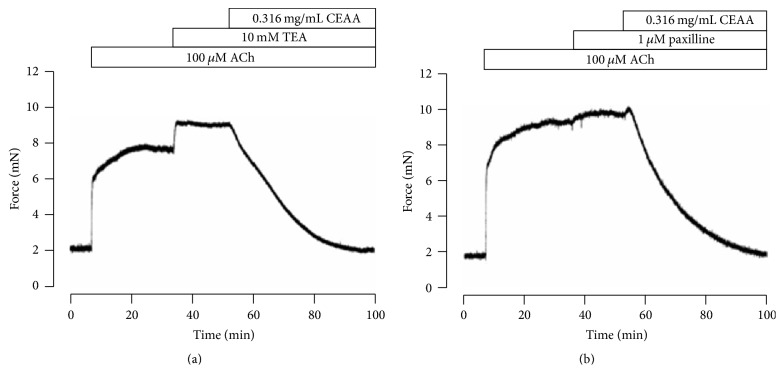
K^+^ channel blockers enhance ACh-induced contraction. (a) TEA-Cl, a nonselective blocker of K^+^ channels, significantly potentiated ACh-induced contractions, which were eliminated completely by CEAA. (b) Paxilline, a selective blocker of BK channels, noticeably potentiated ACh-induced contraction, which was then inhibited by CEAA.

K^+^ channels are ubiquitously distributed in smooth muscle cells and play a key role in regulating smooth muscle tone. We then tested whether K^+^ channels were involved in CEAA-induced relaxant effects. As shown in Figures 6(a) and 6(b), both TEA (a nonselective blocker of K^+^ channels) and paxilline (a selective blocker of BK channels) enhanced ACh-induced contractions, which were relaxed drastically by CEAA.

### 3.5. CEAA Augmented BK Currents by Increasing the Opening Probability

BK channels are highly expressed in smooth muscle cells and play a key role in regulating smooth muscle tone by stabilizing the cell membrane at negative potentials [22]. The following experiments were conducted to determine whether it was BK channels that were involved in CEAA-induced relaxation.

**Figure 7:**
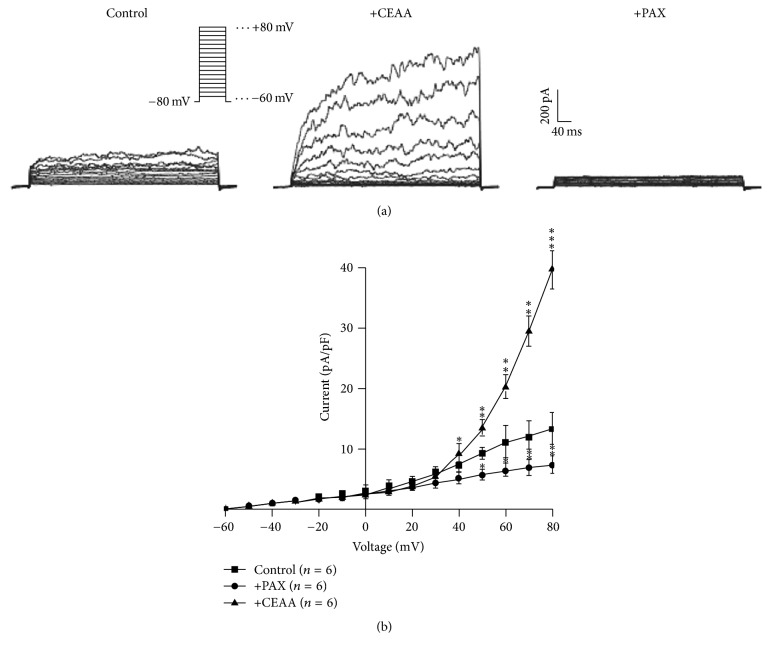
Stimulatory effects of CEAA on whole-cell BK outward currents in ASMCs. (a) Examples of whole-cell BK current traces in the absence (left) or presence (middle) of CEAA (0.316 mg/ml) and the application of paxilline (right). (b) Mean relationship between current and voltage of outward currents at the end of voltage pulses before and after application of CEAA (0.316 mg/ml), ^*∗*^*P* < 0.05; ^*∗∗*^*P* < 0.01; ^*∗∗∗*^*P* < 0.001.

In whole-cell patch-clamp configuration, depolarizing voltage steps with 10 mV increments were applied from a holding potential of −80 mV to + 80 mV to elicit BK currents. As shown in Figure 7(a), CEAA markedly augmented the outward BK current amplitude when the depolarizing pulses from +40 to +80 mV were applied, and paxilline almost fully inhibited these BK currents. The current-voltage (*I-V*) relationship for the BK channels was plotted in the absence and presence of CEAA, as shown in Figure 7(b). We further observe the reversibility of the effect of CEAA on BK currents. As shown in Figure S2, when CEAA is washing out, the augmented BK currents partially recovered by 53.9 ± 4.8%. We also observed that, under paxilline-preincubated conditions, CEAA only slightly increased BK currents (Figure S3).

**Figure 8:**
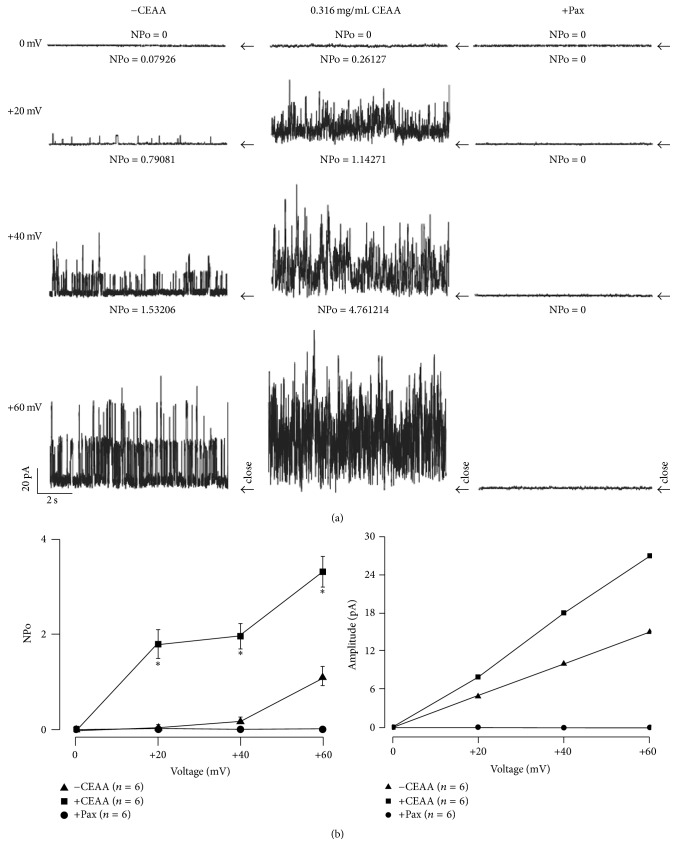
Effect of CEAA on the* I/V* relationship of BK channels in single channel recording in ASMCs. (a) Examples of BK channels in the absence (left) and the presence (middle) of CEAA (0.316 mg/ml) and the application of paxilline (right) measured from an outside-out patch recording at various membrane potentials from 0 to +60 mV with 20 mV increments. Arrows indicate the baseline (all channels in nonpermeant states). The upward current on baseline indicates channel opening. (b) Averaged NPo ratios/voltage and* I/V* relationships of BK channels were plotted in the absence and presence of CEAA (0.316 mg/ml), ^*∗*^*P* < 0.05; ^*∗∗*^*P* < 0.01; ^*∗∗∗*^*P* < 0.001.

To obtain more detailed information on the contribution of BK channels on CEAA-mediated relaxation, single channel recordings were carried out with an outside-out configuration. As shown in Figure 8(a), the single channel BK current traces were recorded (left), and the single channel current amplitude was 15 pA at +60 mV. The application of CEAA dramatically enhanced the activity of the BK channels (middle); paxilline eliminated the activity of the BK channels (right). Opening probability-voltage curve (Figure 8(b), left) and* I-V* curve (Figure 8(b), right) were plotted. The augmented effect of CEAA on whole-cell BK current amplitude was restricted to BK channel opening probability, but the single channel conductance remained unchanged. These effects underscored the potent functions of BK currents in CEAA-mediated relaxation.

## 4. Discussion

The current* in vitro* study demonstrated, for the first time, that, in addition to its antimalaria effect, the well-known traditional Chinese herbal drug* Artemisia annua *L. significantly inhibited ACh/high K^+^-evoked contractile responses of TRs from mice and displayed potent relaxant effects on ACh-prestimulated airway smooth muscle; the suppressed VDCC-mediated Ca^2+^ influx and enhanced activity of BK channels may be involved in the underlying mechanisms of CEAA's relaxant effects.

Airway smooth muscle contraction is generally triggered by a rise in cytosolic free Ca^2+^ concentration ([Ca^2+^]_*i*_), which is regulated by either neurotransmitters coupling of receptors that induce contraction or by membrane depolarization itself in a process known as electromechanical coupling, such as high K^+^ stimuli [23, 24]. High K^+^-induced contraction is believed to rely on cell surface VDCCs, which are activated by cell membrane depolarization and cause an increase in [Ca^2+^]_*i*_ [25]. ACh is one of the most important neurotransmitters to determine the airway tone, and it exerts its contractile effects through multiple pathways, including activating VDCCs and suppressing BK channels [21, 26–28]. Both increased contractility and impaired/incomplete relaxation of airway ASMCs contribute to the hyperresponsiveness observed in asthma [29]. Thus, antagonizing ACh-induced VDCC activation and augmenting BK channels may lead to ASM relaxation. In fact, antagonizing ACh-induced stimulation is still one of the major antiasthma strategies. For example, *β*_2_ AR-agonists, the first-line bronchodilators, relaxed the ASM via antipodal pathways of ACh-induced contraction to exert antiasthma effects [30]. Although the antiasthma effects of these *β*_2_ ARs-agonists have been demonstrated, the side-effects and high recurrence rates limit the use of these therapeutics in patients [3].

We first employed two stimuli, high K^+^ and ACh, to elicit mouse TRs contractions and then assessed CEAA's effects on these contractions. CEAA could markedly antagonize both ACh and high K^+^-elicited TR contractions in mice, and the maximum relaxant efficiency reached almost 100% (Figures 1 and 4). These data demonstrated that CEAA possesses a relaxant potency against contractions induced by ACh/high K^+^ stimulation.

We further investigated the underlying mechanism of the CEAA-mediated relaxant effects. The experiments were conducted under extracellular 0 Ca^2+^/2 mM Ca^2+^ conditions. We first identified the pathways that were involved in the high K^+^-induced contractions. In our study, high K^+^-induced contractions were completely abolished in a Ca^2+^-free medium (Figure 2(a)), suggesting that this type of contraction may be dependent on Ca^2+^ influx through VDCCs [31]. Meanwhile, CEAA almost fully blocked VDCC currents on ASMCs (Figure 3). These data indicated that CEAA relaxed high K^+^-induced contraction by blocking VDCC-mediated Ca^2+^ influx.

We then explored the pathways involved in CEAA-mediated relaxation of ACh-induced contractions. Both VDCCs and BK channels contributed to ACh-induced contractions [27]. Did CEAA mediate its relaxant effects on ACh-induced contractions by regulating these channels? We observed that nifedipine, a selective blocker of VDCCs, partially inhibited ACh-induced sustained contractions under 2 mM Ca^2+^ conditions but had no effect on ACh-induced transient contractions under 0 Ca^2+^ conditions (Figures 5(a), 5(b), and 5(c)). These results suggested that VDCCs were responsible for extracellular Ca^2+^ influx-triggered long-lasting contractions, but not intracellular Ca^2+^ releasing-induced transient contractions. However, CEAA almost eliminated both of the two types of contractions, which suggested that the relaxant effects of CEAA depended on blocking both the extracellular Ca^2+^ influx-mediated by VDCCs and intracellular Ca^2+^ releasing. Paxilline, a selective blocker of BK channels, mildly potentiated ACh-induced contraction, indicating that BK channels might be involved in CEAA-mediated relaxant effects (Figure 6). The subsequent electrophysiological experiments were consistent with these observations, as CEAA completely inhibited VDCC-mediated currents (Figure 3) and noticeably augmented BK currents (Figure 7). The inhibited VDCC currents could directly limit the Ca^2+^ influx to attenuate ACh-induced contractions. CEAA-mediated BK current augmentation would generate stronger outward K^+^ currents, which opposed the depolarization-induced Ca^2+^ influx to relax smooth muscle contraction, indicating that BK channels are an important negative feedback system. Moreover, the augmented effect of CEAA on BK channels lasted a long time, as washing out of CEAA could only partially recover the BK currents (Figure S2). Therefore, it is reasonable that augmentation of BK channel activity favors the relaxation of mice TRs. The single channel analysis further demonstrated that CEAA potentiated the whole-cell BK currents by increasing the opening probability but not changing the single channel conductance (Figure 8). Since BK channels play important roles in shaping the excitability and firing patterns of cells, it is not surprising that their opening probability is easily targeted by many pharmacological compounds [32]. However, under paxilline-preincubated conditions, CEAA could only slightly increase BK currents. These data, taken together, demonstrated that CEAA relaxed effectively ACh/high K^+^-induced precontraction mainly by inhibiting VDCC-mediated Ca^2+^ influx, but the augmented BK currents by CEAA might play a less important role. In addition, other pathways except VDCCs and BK channels might be involved in CEAA-mediated relaxation.

Our results, for the first time, showed that CEAA exhibited strong relaxant effects on ACh/high K^+^-induced precontraction. Further experiments with various ion channel blockers showed that the effect was mediated by inhibiting Ca^2+^-permeable ion channels and enhancing BK channels.

## 5. Conclusion

In summary, the present study demonstrated that an* Artemisia annua *extract can relax ASM contractions. This relaxant effect may be associated with blocking VDCC-mediated Ca^2+^ influx and enhancing BK-mediated K^+^ conductance. Unknown pathways might also be involved in CEAA-mediated relaxation in addition to VDCCs and BK channels. Our findings provide evidence for the potential development of* Artemisia annua *as a novel bronchodilator to treat obstructive airway diseases, such as asthma and COPD.

## Supplementary Material

Figure S1: The washing our experiments of CEAA on ACh/high K+-induced contractions or BK currents.
Figure S2: Effects of CEAA on BK currents in ASMCs under paxilline-preincubated conditions.
Figure S3: Stimulatory effects of CEAA on whole-cell BK outward currents under paxilline-preincubated conditions in ASMCs. A example of whole-cell BK current traces in the absence (left) or presence (middle) of paxilline and the application of CEAA (0.316 mg/ml) (right).

## List of Abbreviations

ACh: Acetylcholine
CEAA: Chloroform extract of* Artemisia annua *L.
TRs: Tracheal rings
ASM: Airway smooth muscle
ASMCs: Airway smooth muscle cells
COPD: Chronic obstructive pulmonary disease
*β*_2_ AR: *β*_2_ adrenergic receptor
SPF: Specific pathogen-free
TEA: Tetraethylammonium
VDCCs: Voltage-dependent Ca^2+^ channels
BK channels: Large conductance voltage- and calcium-activated potassium channels.
