# Supplementary material for: Chloroform Extract of Artemisia annua L. Relaxes Mouse Airway Smooth Muscle
Source: Evid Based Complement Alternat Med. 2017 Nov 12;2017:9870414. doi: 10.1155/2017/9870414 (PMC5702405; doi:10.1155/2017/9870414)
Supplement: Supplementary file 1 — Figure S1: The washing our experiments of CEAA on ACh/high K+-induced contractions or BK currents. Figure S2: Effects of CEAA on BK currents in ASMCs under paxilline-preincubated conditions. Figure S3: Stimulatory effects of CEAA on whole-cell BK outward currents under paxilline-preincubated conditions in ASMCs. A example of whole-cell BK current traces in the absence (left) or presence (middle) of paxilline and the application of CEAA (0.316 mg/ml) (right). [file 9870414.f1.pdf]

Supplementary Figures

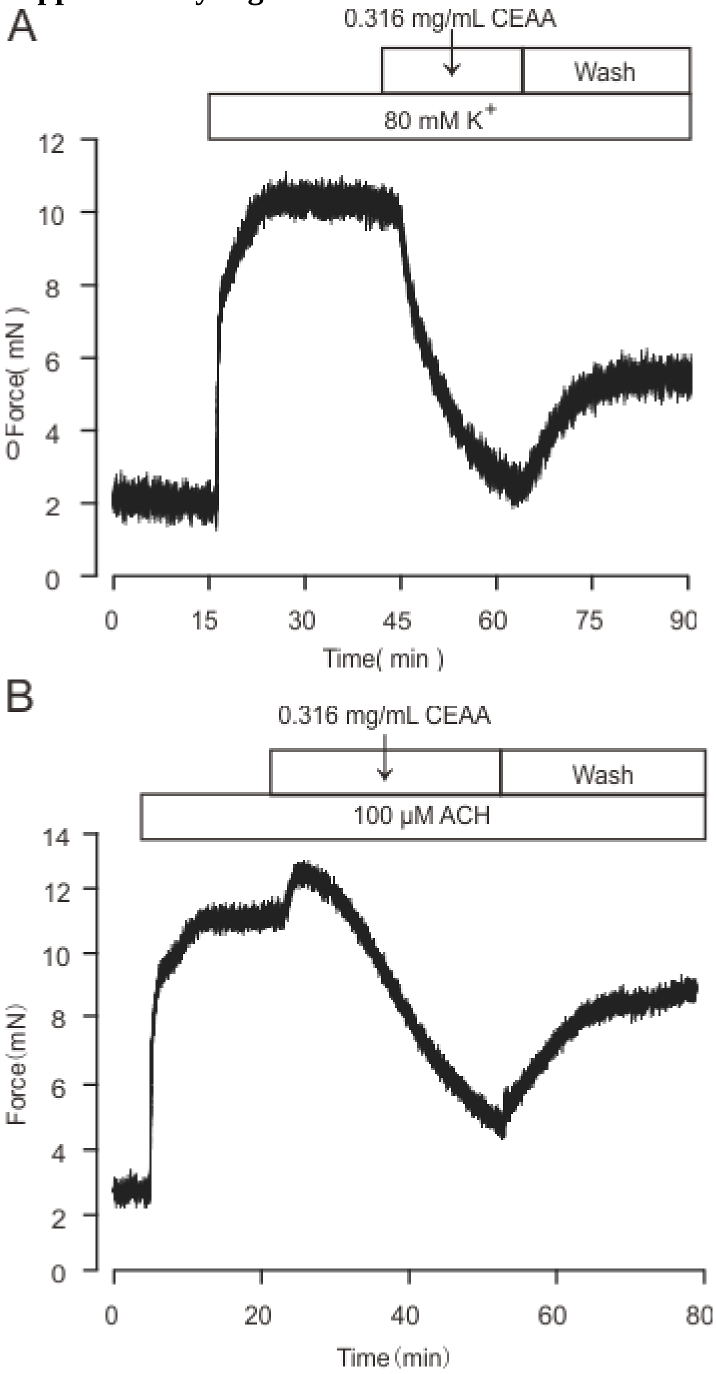

Fig. S1. The relaxant effects of CEAA on ACh/high K^+-induced contractions were partially reversible. A. After washing out of CEAA, high K^+-induced contraction recovered by 35.9%±3.5%. B. After washing out of CEAA, ACh-induced contraction recovered by 45.8%±1.2%.

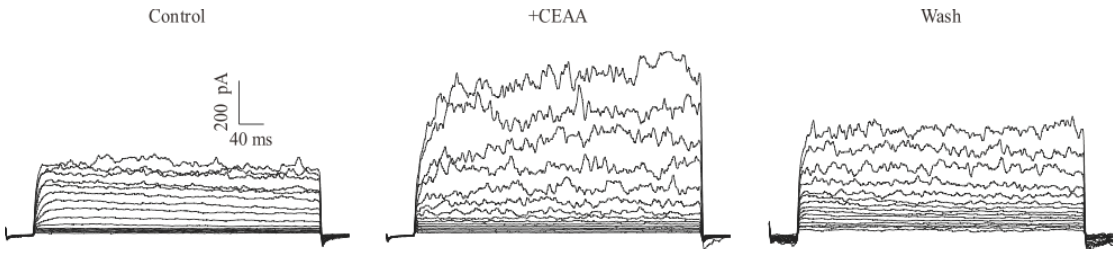

Fig. S2. The enhanced BK currents by application of CEAA partially recovered with washout. A Example of whole-cell BK current traces in the absence (left) or presence (middle) of CEAA (0.316 mg/ml) and washing out of CEAA (right).

.

.
.

.

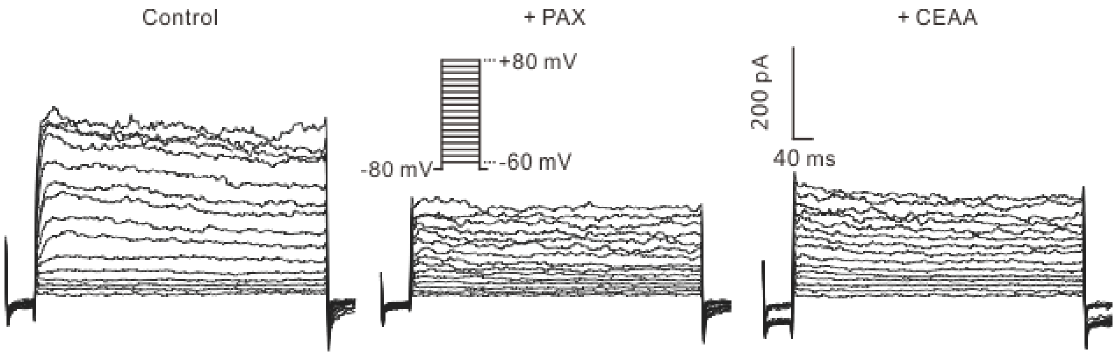

Fig. S3. Stimulatory effects of CEAA on whole-cell BK outward currents in ASMCs under paxilline-preincubated conditions. A example of whole-cell BK current traces in the absence (left) or presence (middle) of paxilline and the application of CEAA (0.316 mg/ml) (right).
